# Development of Long Noncoding RNA-Based Strategies to Modulate Tissue Vascularization

**DOI:** 10.1016/j.jacc.2015.07.081

**Published:** 2015-11-03

**Authors:** Jan Fiedler, Kaja Breckwoldt, Christian W. Remmele, Dorothee Hartmann, Marcus Dittrich, Angelika Pfanne, Annette Just, Ke Xiao, Meik Kunz, Tobias Müller, Arne Hansen, Robert Geffers, Thomas Dandekar, Thomas Eschenhagen, Thomas Thum

**Affiliations:** ∗ Institute of Molecular and Translational Therapeutic Strategies, Hannover Medical School, Hannover, Germany; † Integrated Research and Treatment Center Transplantation, Hannover Medical School, Hannover, Germany; ‡ Department of Experimental Pharmacology and Toxicology, University Medical Center Hamburg, Eppendorf, Germany; § DZHK (German Centre for Cardiovascular Research), Partner Site, Hamburg/Kiel/Lübeck, Germany; ‖ Department of Bioinformatics, University of Würzburg, Würzburg, Germany; ¶ Institute of Human Genetics, University of Würzburg, Würzburg, Germany; # Helmholtz Centre for Infection Research, Braunschweig, Germany; ∗∗ National Heart and Lung Institute, Imperial College London, London, United Kingdom; †† REBIRTH Excellence Cluster, Hannover Medical School, Hannover, Germany

**Keywords:** angiogenesis, endothelial cell biology, hypoxia, lncRNA, EC, endothelial cell, EHT, engineered heart tissue, GFP, green fluorescent protein, HIF1α, hypoxia-inducible factor 1α, HUVEC, human umbilical vein endothelial cell, lincRNA, long intergenic noncoding ribonucleic acid, lncRNA, long noncoding ribonucleic acid, miR, mature micro–ribonucleic acid, miRNA, micro–ribonucleic acid, mRNA, messenger ribonucleic acid, RNA, ribonucleic acid, RNA-seq, ribonucleic acid sequencing, siRNA, small interfering ribonucleic acid, VEGF, vascular endothelial growth factor

## Abstract

**Background:**

Long noncoding ribonucleic acids (lncRNAs) are a subclass of regulatory noncoding ribonucleic acids for which expression and function in human endothelial cells and angiogenic processes is not well studied.

**Objectives:**

The authors discovered hypoxia-sensitive human lncRNAs via next-generation ribonucleic acid sequencing and microarray approaches. To address their functional importance in angiogenic processes, several endothelial lncRNAs were characterized for their angiogenic characteristics in vitro and ex vivo.

**Methods:**

Ribonucleic acid sequencing and microarray-derived data showed specific endothelial lncRNA expression changes after hypoxia. Validation experiments confirmed strong hypoxia-dependent activation of 2 intergenic lncRNAs: LINC00323 and MIR503HG.

**Results:**

Silencing of these lncRNA transcripts led to angiogenic defects, including repression of growth factor signaling and/or the key endothelial transcription factor GATA2. Endothelial loss of these hypoxia-driven lncRNAs impaired cell-cycle control and inhibited capillary formation. The potential clinical importance of these endothelial lncRNAs to vascular structural integrity was demonstrated in an ex vivo model of human induced pluripotent stem cell–based engineered heart tissue.

**Conclusions:**

The authors report an expression atlas of human hypoxia-sensitive lncRNAs and identified 2 lncRNAs with important functions to sustain endothelial cell biology. LncRNAs hold great promise to serve as important future therapeutic targets of cardiovascular disease.

The identification of the expression and function of cardiovascular noncoding ribonucleic acids (RNAs), such as microRNAs (miRNAs) (or “miR,” which refers to the mature form of miRNA), has opened a new avenue of genome-regulatory paradigms. (The prefix “miR” usually is followed by a hyphen and a number, the latter often indicating order of naming.)

Numerous reports have proved that single miRs have a master role in the regulation of cardiovascular pathophysiology and serve as interesting treatment targets 1, 2. Of importance, miRs are already used in diagnostic as well as therapeutic approaches 3, 4. Recently, the more heterogeneous RNA subclass of long noncoding RNAs (lncRNAs) has become the focus of intense research. LncRNAs are defined as non-protein-coding RNAs >200 nucleotides in length. Their genomic localization can either be in sense or antisense to coding genes or intergenic 5, 6.

The current annotation of lncRNAs within databases is a very dynamic process because of actively increasing knowledge in this field (7). In contrast to miRNAs, whose functional relevance is due mainly to targeting of partial complementary messenger RNA (mRNA), lncRNA-based mechanisms are more diverse and also dependent on their localization within the nuclear or cytosolic compartment 8, 9. In vivo, loss-of-function analysis has revealed the crucial importance of certain lncRNAs during cardiac wall and cardiac lineage development 10, 11. An MYH7-encoded lncRNA, Myhrt, was identified to regulate cardiac function and be a valuable therapeutic target to counteract cardiac hypertrophy (12). Expression of function of some other lncRNAs in the vasculature has been described before; for instance, the control of endothelial cell (EC) homeostasis and junction contacts was demonstrated to be dependent on antagonism of Tie-1 mRNA via Tie-1 antisense lncRNA in vivo (13). In line, Tie1 antisense lncRNA was increased in patients with vascular anomalies, indicating therapeutic potential. Another antisense lncRNA, SENCR, was identified in human artery smooth muscle cells using RNA sequencing (RNA-seq) (14). SENCR knockdown triggered a cascade of antimigratory events in this type of vascular cell.

Very recently, endothelial lncRNA MIAT was found to be induced in diabetic conditions (15). Therapeutic intervention with MIAT improved microvascular dysfunction in a diabetic disease model in vivo via interference with binding of MIAT toward vascular endothelial growth factor (VEGF) and miR-150-5p. The endothelial-enriched lncRNA MALAT1 was also shown to control the migration and sprouting of ECs (16). However, none of the aforementioned lncRNAs was specifically investigated in the setting of endothelial hypoxia.

LncRNAs may also enter the circulation to form a basis for new diagnostic and prognostic strategic approaches to cardiovascular disease 17, 18. Hypoxia is a major trigger of angiogenic events in ECs (19). In strong contrast to miRNAs, current knowledge about the expression and function of lncRNAs in human ECs is nascent. A direct long intergenic noncoding RNA (lincRNA)–p21 mediated mechanism of hypoxia-inducible factor 1α (HIF1α) stabilization and a potential role in angiogenic events has been demonstrated but, so far, in cancer cells only (20).

In this work, we globally studied whether lncRNAs are differentially regulated in human ECs through hypoxia using RNA-seq and microarray approaches and applied gain- and loss-of-function studies for selected novel endothelial lncRNAs in vitro and ex vivo to study their functional importance for endothelial biology. Thus, the aim of this study was to provide an overview of hypoxia-sensitive functional lncRNAs to discover new entry points for the development of lncRNA-based strategies to modulate tissue vascularization.

## Methods

We used 2 μg of total RNA from human umbilical vein ECs (HUVECs) cultured under normoxic or hypoxic conditions for 24 h and subjected to RNA-seq analysis (n = 3). After extracting the total RNA from the samples, ribosomal RNA was removed from the total RNA. By using the fragmentation buffer, the left RNA was fragmented into short fragments, then the first strand complementary deoxyribonucleic acid was synthesized by random hexamer-primer using the left RNA fragments as templates. Buffer, nucleotide triphosphates containing deoxyribose, ribonuclease H, and deoxyribonucleic acid polymerase I were added to synthesize the second strand. The double-strand complementary deoxyribonucleic acid was purified using the QiaQuick polymerase chain reaction extraction kit (Qiagen, Venlo, the Netherlands) and washed with elution buffer for end repair and poly(A) addition. Finally, sequencing adaptors were ligated to the fragments, which were purified by agarose gel electrophoresis and enriched by polymerase chain reaction amplification. The library products were sequenced using the HiSeq 2000 (Illumina, San Diego, California).

A detailed methods section is available in the Online Appendix.

## Results

### Signature of endothelial lncRNA expression during hypoxia

To identify hypoxia-sensitive endothelial lncRNAs, HUVECs were first subjected to 24 h of hypoxia. In preliminary experiments, we found this time point optimal for identifying regulations of a vast majority of lncRNAs (data not shown). Hypoxia-sensitive noncoding RNA expression was detected via 2 approaches, microarray- and RNA-seq-based techniques. We first focused on the microarray-derived noncoding RNA dataset to search for lncRNAs using the following criteria: minimal 4-fold deregulation and intergenic lncRNA localization. By this approach, we identified LINC00323 (lnc-DSCAM-1) to be the strongest (∼8-fold) up-regulated lincRNA after hypoxia (probe uc002yyv) (Online Table 1).

**Figure 1:**
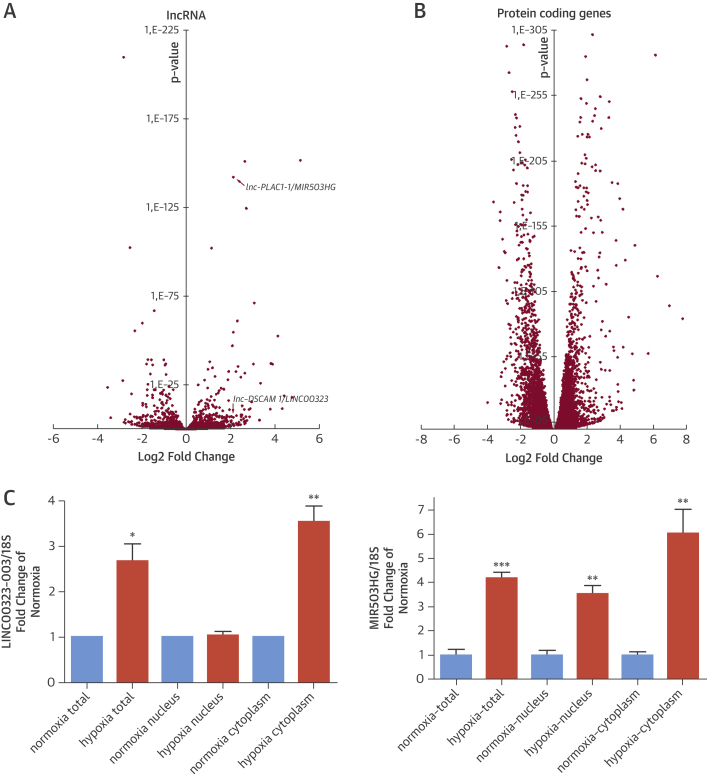
RNA-Sequencing and Microarray Application to Identify Hypoxia-Sensitive Noncoding RNAs in HUVECs **(A)** Differentially expressed long noncoding ribonucleic acids (lncRNAs) are represented in this volcano plot of hypoxic versus normoxic human umbilical vein endothelial cells (HUVECs) (fold change) from ribonucleic acid (RNA) sequencing analysis. MIR503HG/lnc-PLAC1-1 and LINC00323/lnc-DSCAM-1 are marked with **red arrows**. The x-axis shows log_2_-transformed fold change; the y-axis shows adjusted p values (Online Table 2). **(B)** Differentially expressed messenger RNAs (mRNAs) for coding genes are shown in a volcano plot of hypoxic versus normoxic HUVECs from RNA sequencing. The x-axis shows log_2_-transformed fold change; the y-axis shows adjusted p values (Online Table 2). **(C)** Long intergenic noncoding ribonucleic acids (lincRNAs) LINC00323-003 and MIR503HG in fractionated RNA (total, nuclear, and cytoplasmic) from HUVECs subjected to normoxia and hypoxia are validated. Fold change of normoxia (set to 1) is plotted. Qualitative polymerase chain reaction analysis for LINC00323-003 applying ΔΔCt-method, for MIR503HG applying standard curve analysis (n = 3/4 experiments with 3 technical replicates). *p < 0.05; **p < 0.01; ***p < 0.001.

RNA-seq data identified 773 (403 up, 370 down) significantly deregulated lncRNAs (Figure 1A, Online Table 2). The analysis of RNA-seq data for protein-coding genes of HUVECs cultured under normoxic or hypoxic conditions showed 8,156 (4,032 up, 4,124 down) significantly deregulated mRNAs (Figure 1B, Online Table 2). The log_2_-fold expression changes derived from the 2 different technologies were significantly correlated both for lncRNAs (Pearson correlation coefficient = 0.43, p < 2.2 × 10^−16^) and for protein-coding genes (Pearson correlation coefficient = 0.74, p < 2.2 × 10^−16^) (Online Figure 1). RNA-seq also confirmed enhanced expression of LINC00323 (lnc-DSCAM-1) under hypoxia (Online Table 2).

In addition, from the RNA-seq data, we identified another hypoxia-sensitive lncRNA, MIR503HG (lnc-PLAC1-1), on the basis of the following criteria: 1) minimal 4-fold up-regulation; 2) base mean >1,000; and 3) intergenic annotation. On the basis of these stringent criteria, we identified lnc-MST1P9-2 and lnc-PLAC1-1 as high propriety candidates but omitted lnc-MST1P9-2 from further studies because of potential protein-coding properties. Subsequent validation experiments confirmed the hypoxia-dependent up-regulation of the previous 2 array- or RNA-seq-derived lincRNAs, LINC00323 and MIR503HG (Figure 1C and Online Figure 1 for other LINC00323 transcript isoforms). Cell compartment–specific RNA analysis revealed hypoxia-dependent enhanced cytoplasmic LINC00323-003 expression, whereas MIR503HG was increased both in the nuclear and cytoplasmic compartments (Figure 1C). High purity of subcellular compartment preparations was proved by screening expression of the nuclear enriched lncRNA XIST (Online Figure 1). Endothelial up-regulation of these 2 lncRNAs by hypoxia was cell-type specific, as this was not seen in human cardiac fibroblasts or human aortic smooth muscle cells exposed to hypoxia (Online Figure 1). The 2 lncRNAs were considerably expressed in ECs and vascularized organs, such as kidneys (data not shown). We next sought to identify potential functional relevance for the identified lncRNA candidates in human ECs.

### LINC00323-003 and angiogenic functions

**Figure 2:**
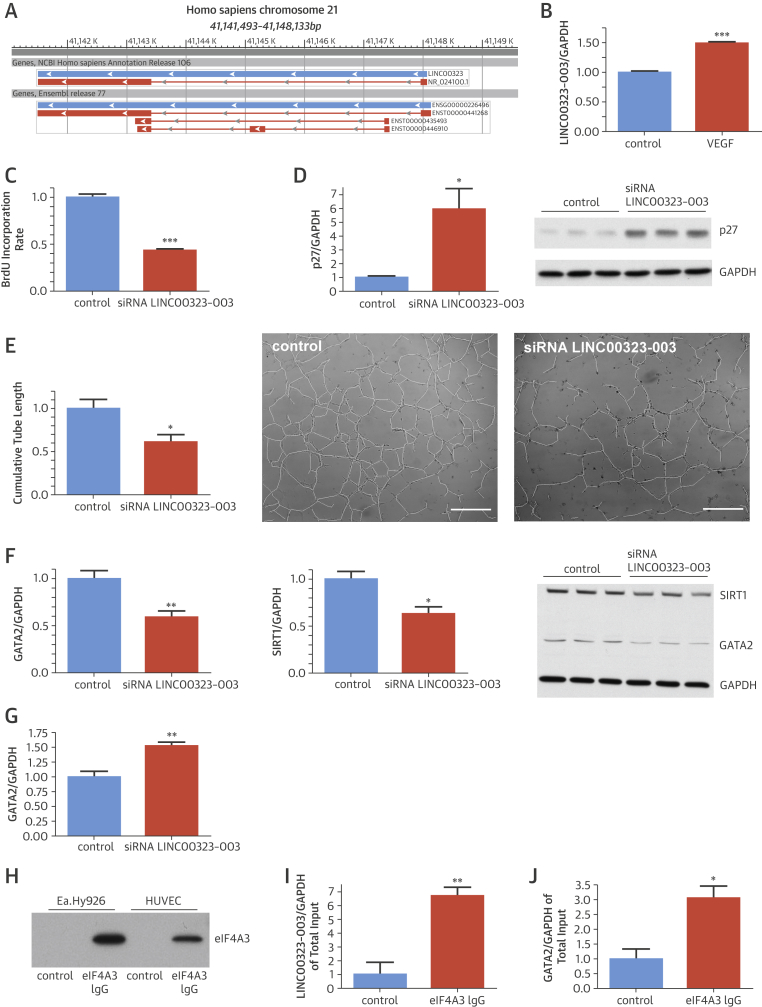
Endothelial LINCRNA LINC00323-003 Expression Is Crucial for Endothelial Cellular Function **(A)** Genomic localization of LINC00323-003, adapted from National Center for Biotechnology Information Map Viewer. **(B)** Human umbilical vein endothelial cells (HUVECs) treated with vascular endothelial growth factor (VEGF) (50 ng/ml) for 24 h (n = 3 experiments). **(C)** Liposomal-based small interfering ribonucleic acid (siRNA) transfection against LINC00323-003 decreases bromodeoxyuridine (BrdU) incorporation rate measured by BrdU enzyme linked immunosorbent assay (n = 3 experiments). **(D)** The cell-cycle inhibitor p27 is increased in LINC00323-003 knockdown HUVECs (n = 3 experiments). **(E)** Matrigel-based tube formation assay demonstrates less capillary formation in LINC00323-003-deficient HUVECs. Scale bar = 500 μm (n = 3 experiments). **(F)** GATA2 and sirtuin I (SIRT1) are reduced in siRNA LINC00323-003 transfected HUVECs (n = 4 experiments). **(G)** GATA2 messenger ribonucleic acid (mRNA) is enhanced after LINC00323-003 knockdown (n = 4 experiments). **(H)** Immunoprecipitation (IP) for eIF4A3 in human endothelial cells Ea.Hy926 and HUVEC. Enrichment for eIF4A3 is seen in the fraction using eIF4A3-specific antibodies for IP. **(I)** eIF4A3-IP from human Ea.Hy926 cells and subsequent ribonucleic acid (RNA) isolation reveals enrichment of LINC00323-003 RNA via qualitative polymerase chain reaction (n = 3 experiments). **(J)** GATA2 mRNA associates with eIF4A3 in Ea.Hy926 cells (n = 3 experiments). All experiments were conducted with 3 technical replicates. *p < 0.05; **p < 0.01; ***p < 0.001. IgG = immunoglobulin G.

LINC00323 is encoded on chromosome 21 and has 3 transcript isoforms 21, 22 according to the lncRNA database LNCipedia and the Ensembl Genome Browser (Figure 2A). We focused on LINC00323 transcript variant 3, as a small interfering RNA (siRNA) approach had obvious effects on EC biology to reduce cell number in vitro. Next to proangiogenic hypoxic signaling, endothelial VEGF treatment also increased LINC00323-003 expression (Figure 2B). Multiple in vitro assays were performed to clarify the functional relevance of endothelial LINC00323-003 expression. Because LINC00323-003 was detectable in nuclear and cytoplasmatic compartments, we used both antisense-mediated siRNA (targeting cytoplasmic lncRNAs) and GapmeR (targeting nuclear lncRNAs) approaches in HUVECs. Both approaches in a transgenic LINC00323-003 overexpressing EC line documented potent lncRNA silencing (Online Figure 2). Liposomal-based control GapmeR–fluorescein transfection in ECs led to an accumulation in the nucleus (Online Figure 2). Titration experiments for the siRNA chemistry showed detrimental effects on EC viability in a range of 1 to 100 nmol/l siRNA, whereas application of 100 pmol/l of siRNA exhibited no significant effect (Online Figure 2). Further siRNA experiments were thus performed with 1 nmol/L of siRNA to reduce potential off-target effects. Both siRNA- and GapmeR-mediated LINC00323-003 silencing led to an inhibition of human EC proliferation (Figure 2C, Online Figure 2), suggesting cytoplasmic and nuclear LINC00323-003 expression to be critical for endothelial proliferation. In line, LINC00323-003 silencing increased the ratio of binucleic to total cells and led to an increase in EC size, suggesting proliferation defects (Online Figures 2 and 3). Accordingly, the cell-cycle inhibitors p21 and p27 were up-regulated, whereas cell-cycle progression-relevant cyclin-dependent kinases 1 and 2 were reduced upon endothelial LINC00323-003 silencing (Figure 2D, Online Figure 3). Migration capacity was also strongly attenuated by LINC00323-003 loss (Online Figure 3). Angiogenic capacity tested by capillary tube formation assays was significantly deteriorated upon LINC00323-003 silencing (Figure 2E). This was paralleled by a repression of proangiogenic VEGFb and induction of the angiogenesis inhibitor thrombospondin (Online Figure 3). Loss of LINC00323-003 additionally inhibited proliferation-important extracellular signal–regulated kinase phosphorylation, and both led to a repression of the key endothelial transcription factor GATA2 and proangiogenic sirtuin I (Online Figure 3, Figure 2F). Indeed, part of the detrimental actions of LINC00323-003 were mediated through GATA2 silencing, as genetic overexpression of a functional GATA2 construct attenuated the LINC00323-003 actions on ECs (Online Figure 3). Interestingly, LINC00323-003 silencing reduced GATA2 protein but not mRNA levels (Figure 2G), suggesting a post-translational mechanism. Using starBase 23, 24, a bioinformatic-based identification system, we found potential protein candidates interacting with LINC00323-003 that are also involved in mRNA translation and identified the translation initiation factor eIF4A3. Indeed, we were able to immunoprecipitate eIF4A3 in human ECs (Figure 2H). RNA immunoprecipitation experiments in a stable LINC00323-003-overexpression EC line revealed significant enrichment of LINC00323-003 with immunoprecipitated eIF4A3 compared with controls (Figure 2I). In addition, GATA2 mRNA was found to be associated with eIF4A3 (Figure 2J). This suggests a scaffold function of LINC00323-003 to bind proteins, such as the translation initiation factor eIF4A3 (Central Illustration). LINC00323-003 silencing also caused impaired proliferative capacity of human coronary artery ECs, suggesting a broad impact of this lncRNA on various types of ECs (Online Figure 3).

**Central Illustration:**
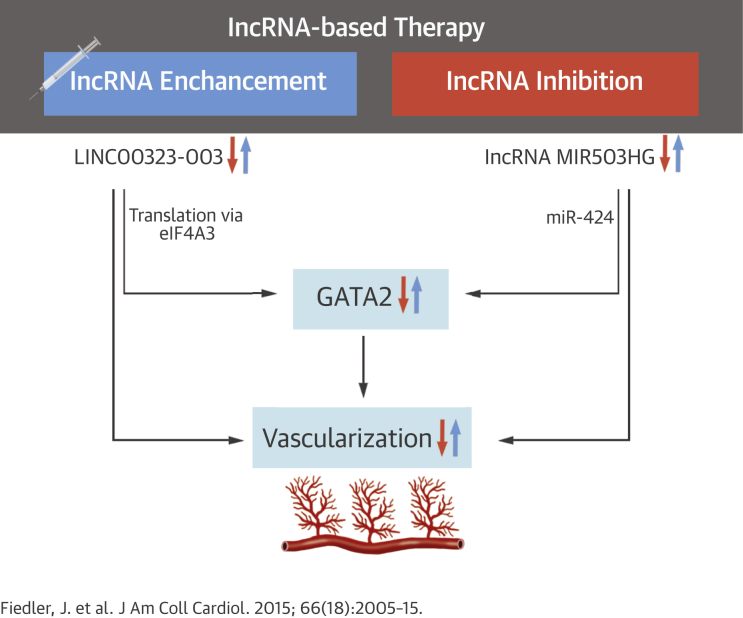
Vascular Importance of Angiogenic Long Noncoding Ribonucleic Acids Summarizing scheme to emphasize vascular importance and therapeutic strategy to modulate LINC00323-003 and MIR503HG expression to improve or to inhibit tissue vascularization. Expression of the long noncoding ribonucleic acids (lncRNAs) LINC00323-003 and MIR503HG is needed to sustain vascular homeostasis and endothelial cell biology; lowering LINC00323-003 and MIR503HG triggers a cascade of antiangiogenic events via repression of GATA2. In the case of LINC00323-003, interaction with eIF4A3 controls GATA2 translation, while lncRNA MIR503HG controls neighboring miR-424 expression.

### Endothelial functions of hypoxia-sensitive lncRNA MIR503HG

**Figure 3:**
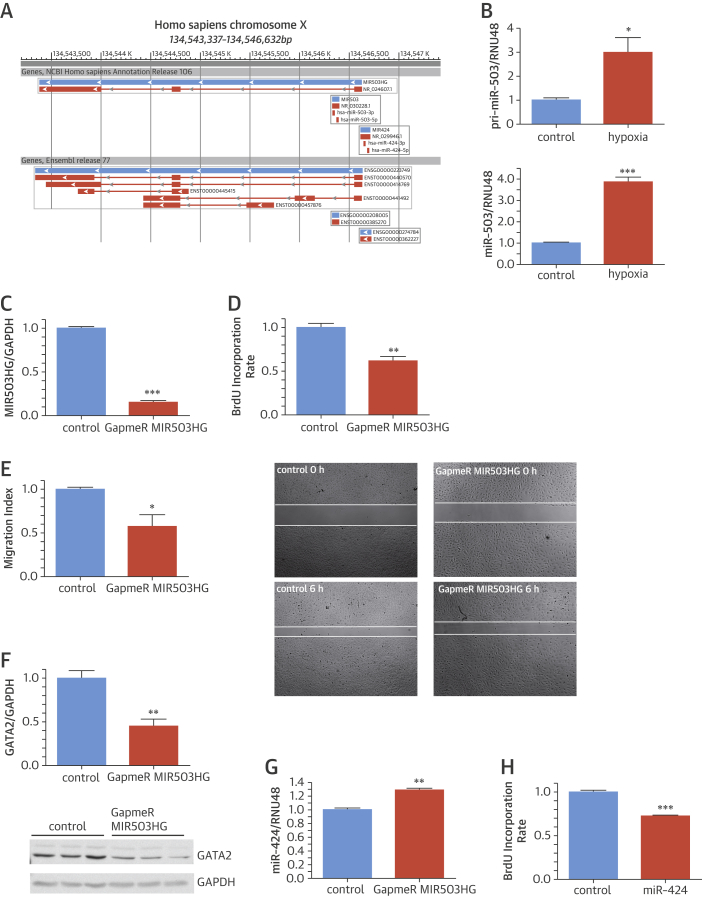
Endothelial LINCRNA MIR503HG Is Transcriptionally Regulated With miR-503 After Hypoxia and its Loss Deteriorates HUVEC Function **(A)** Genomic localization of MIR503HG, adapted from National Center for Biotechnology Information Map Viewer. **(B)** Primary transcript of miR-503 (pri-miR-503) and processed, mature miR-503 are up-regulated after 24 h hypoxia in human umbilical vein endothelial cells (HUVECs) determined by quantitative reverse transcription polymerase chain reaction (qRT-PCR) (n = 4 experiments). **(C)** GapmeR-mediated knockdown of long intergenic noncoding ribonucleic acid (lincRNA) MIR503HG efficiently lowers endogenous expression level in HUVECs detected by qRT-PCR (n = 3 experiments). **(D)** Loss of MIR503HG causes impairment in bromodeoxyuridine (BrdU) incorporation rate monitored by enzyme-linked immunosorbent assay (ELISA) (n = 3 experiments). **(E)** Scratch wound closure is impaired when lincRNA MIR503HG is silenced in HUVECs (n = 3 experiments). **(F)** GapmeR against lincRNA MIR503HG represses GATA2 on protein level (n = 4 experiments). **(G)** Endogenous knockdown of MIR503HG via GapmeR induces endothelial miR-424 expression (n = 3 experiments). **(H)** Synthetic miR-424 overexpression lowers HUVEC proliferation detected via BrdU ELISA (n = 3 experiments). All experiments were conducted with 3 technical replicates. *p < 0.05; **p < 0.01; ***p < 0.001.

The RNA-seq-derived lincRNA MIR503HG is located at chromosome X, harbors a coding sequence of miR-503, and is adjacent to miR-424 (Figure 3A). Indeed, analysis of primary miR-503 and mature miR-503 expression after hypoxia showed a similar increase of expression reflecting same regulatory mechanisms for transcriptional activation (Figure 3B). However, both miR-503 and lincRNA MIR503HG modulations had different effects on EC function; whereas transfection of miR-503 precursors resulted in negative effects on EC viability (Online Figure 4), knockdown of MIR503HG suppressed endothelial proliferation independent of alterations of apoptosis (Figures 3C and 3D, Online Figure 4). MIR503HG silencing via GapmeR technology led to reduced miR-503 expression (Online Figure 4). In contrast, transfection of miR-503 precursors did not alter MIR503HG expression (Online Figure 4), suggesting dependency of miR-503 expression on MIR503HG but not vice versa. Migratory capacity of MIR503HG-deficient ECs was additionally inhibited (Figure 3E). The proliferative and migratory functional defects in ECs after MIR503HG silencing were paralleled by a strong up-regulation of the cell-cycle inhibitor p21 (Online Figure 4). In line with data from LINC00323-003 loss-of-function experiments, silencing of MIR503HG repressed expression of the key angiogenic transcription factor GATA2 (Figure 3F). Additionally, we identified another miRNA, miR-424, genetically neighboring upstream to MIR503HG. Surprisingly, MIR503HG repression up-regulated neighboring miR-424 expression (Figure 3G). Conclusively, this observation supports a *cis*-action of lnc-MIR503HG on neighboring miR-424. MiR-424 was previously described to be antiproliferative in lung arterial ECs; moreover, its overexpression triggers antiangiogenic effects in human ECs (25). Indeed, we were able to validate these findings and found impaired proliferation of human ECs after miR-424 overexpression (Figure 3H). However, no significant effects toward impairment of angiogenesis were observed when analyzing capillary tube formation after MIR503HG silencing (Online Figure 4). Intrinsic modulation in arterial ECs had only minor effects on cellular behavior, suggesting a different impact of MIR503HG in venous and arterial ECs (Online Figure 4).

### Enhanced lincRNA expression improves migratory capacity

To study the role of enhanced lincRNA expression in ECs, the hypoxia-sensitive lincRNAs LINC00323-003 and MIR503HG were both overexpressed by generation of stable green fluorescent protein (GFP)-expressing EC lines (Ea.Hy926) (Online Figure 5). Transgenic Ea.Hy926 constitutively overexpressing LINC00323-003 revealed improved endothelial healing in the scratch wound assay and showed enhanced cytoprotective HMOX1 expression (Online Figure 5). In contrast, no changes in endothelial biology were observed after MIR503HG overexpression (Online Figure 5).

### Translating in vitro cell culture findings into a 3-dimensional ex vivo model

**Figure 4:**
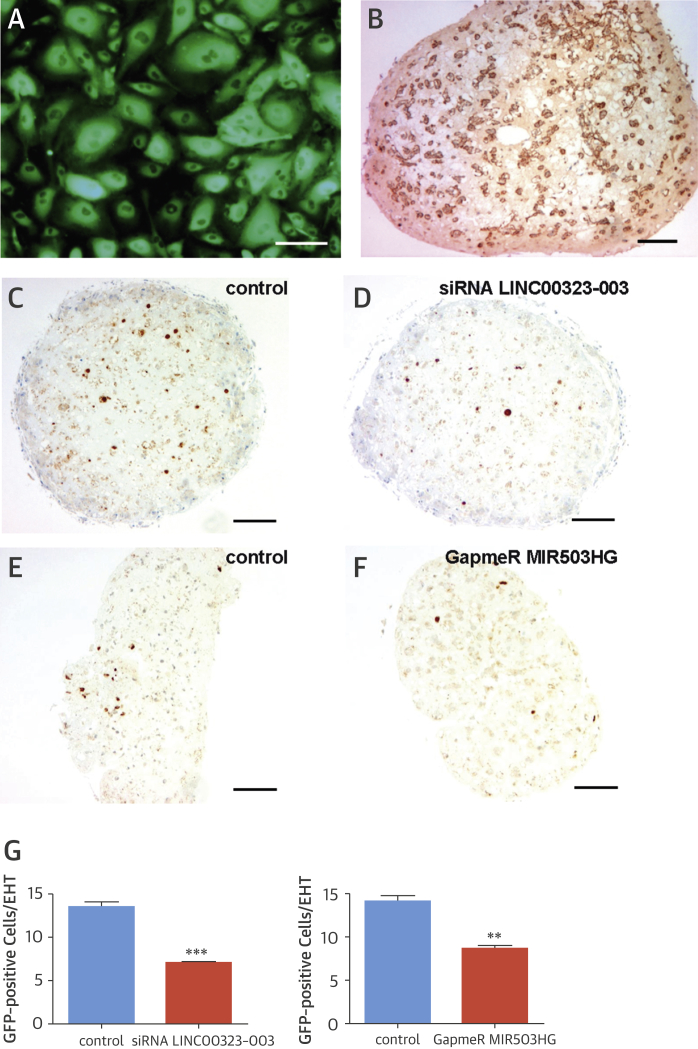
Application of LINCRNA LINC00323-003 and MIR503HG Knockdown Strategy in Human EHT Is Detrimental for Vascularization **(A)** Green fluorescent protein (GFP) fluorescence of lentivirally transduced human umbilical vein endothelial cells (HUVECs). **(B)** Dystrophin staining of cardiac myocytes in cross-sectioned EHT (brown staining of membranes). Scale bar = 100 μm. In GFP staining of cross-sectioned EHTs, showing as intense brown versus slightly brownish nonspecific background staining (scale bar = 100 μm; n = 3 EHTs per group), EHT is transfected with control small interfering ribonucleic acid (siRNA) **(C)**, siRNA LINC00323-003 **(D)**, control GapmeR **(E)**, and GapmeR MIR503HG **(F)**. An analysis of GFP-positive endothelial cells **(G)** reveals larger numbers in controls compared with EHTs that were mixed with LINC00323-003- or MIR503HG-deficient HUVECs (n = 3 experiments). EHT = engineered heart tissue.

To investigate whether the in vitro findings could be validated in a 3-dimensional ex vivo model, we generated human induced pluripotent stem cell–derived engineered heart tissues (EHTs) enriched with GFP-overexpressing HUVECs for easy identification of ECs in such human ex vivo constructs. HUVECs showed 99% purity after blasticidine selection, as confirmed by flow cytometric analysis (data not shown) and GFP fluorescence microscopy (Figure 4A). EHTs were transfected with siRNA LINC00323-003, GapmeR MIR503HG, or scrambled controls on days 5, 10, and 15 of culture, and analyses were performed for EHT development and number of ECs. Dystrophin staining of cross-sectioned EHTs showed a homogenous distribution and high density of cardiomyocytes (Figure 4B), which were similarly observed in all groups (data not shown). Quantification of GFP-expressing HUVECs (Figures 4C to 4G) identified a significantly smaller number of ECs in LINC00323-003 and MIR503HG loss-of-function EHTs compared with control tissue constructs. These data suggest that silencing LINC00323-003 and MIR503HG, not only in vitro but also in an ex vivo human EHT model, impairs endothelial biology and tissue vascularization.

## Discussion

Hypoxia is a strong proangiogenic stimulus and induces VEGF-mediated signaling in ECs (19). The present study provides evidence that lncRNA expression in ECs is strongly altered by hypoxia. We have identified 2 novel lncRNAs, LINC00323-003 and MIR503HG, that are highly sensitive to hypoxia and crucial for endothelial angiogenic characteristics. The mechanisms leading to the hypoxia-mediated regulation of these lncRNAs remain to be determined. To this end, we silenced HIF1α during hypoxic cell culture of HUVECs and tested effects on LINC00323-003 and MIR503HG expression. Silencing HIF1α did not abrogate hypoxia-mediated induction of lncRNAs compared with controls (data not shown). This is suggestive of HIF1α-independent mechanisms of the 2 hypoxia-induced lncRNAs described here and is in line with the Encyclopedia of DNA Elements dataset at the University of California, Santa Cruz, showing no HIF1α-binding sites in promoter regions of these lncRNAs. Importantly, silencing these lnc RNAs impaired the function of ECs blocking proliferative pathways. Both lncRNAs have never been described in endothelial biology, but here we show that they function as crucial factors to control EC behavior.

Key experiments for LINC00323-003 were also validated in arterial ECs, suggesting the importance on ECs in general. Of translational importance, lncRNA knockdown also resulted in impaired vascularization in an ex vivo induced pluripotent stem cell–based 3-dimensional engineered human heart tissue model. This model was used because of weak conservation at the sequence level in nonhuman species. However, a potential mouse homologue for MIR503HG, Gm28730, with about 70% sequence overlap, could be identified. The choice to investigate LINC00323-003 and MIR503HG was dependent on their high deregulation in either microarray or RNA-seq datasets. It is likely that many other thus far uncharacterized lncRNAs also have crucial functions in endothelial biology and should be investigated in future studies; the database of array- and RNA-seq-based lncRNA expression profiles reported here will be helpful in this context.

### Study limitations

Both lncRNAs LINC00323-003 and MIR503HG exerted an important functional relevance for EC biology, making them potential attractive molecular targets in vascular diseases. We have provided initial insight into their interaction partners, such as miRNAs and proteins, but we wish to point out that more in-depth mechanistic studies are needed in the future to fully understand the mode of action of the identified lncRNAs. In general, lncRNAs are less (sequence) conserved among species, thus hampering translational approaches in a relevant in vivo vascular disease setting in other species models. To circumvent this limitation, we applied a model of human induced pluripotent stem cell–based EHT to translate the importance of these 2 lncRNAs in endothelial biology in a more realistic translational scenario.

## Conclusions

We have identified 2 novel hypoxia-sensitive endothelial lncRNAs, LINC00323-003 and MIR503HG, by the use of microarray and next-generation RNA-seq techniques. Functional characterization applying loss- and gain-of-function approaches in cultured ECs revealed an important role for both of these lncRNAs in endothelial biology. Our data enable the future development of novel lncRNA-therapeutic approaches to cardiovascular diseases. More general, our data identify novel strategies based on lncRNA modulation to alter tissue vascularization, with the aim of either enhancing (e.g., in ischemic tissues) or blocking (e.g., in vessel-dependent cancers) vascularization.

Perspectives **COMPETENCY IN MEDICAL KNOWLEDGE:** Angiogenesis is a key mechanism by which organs maintain blood supply, and this has therapeutic implications for amelioration of ischemia in organs or inhibition of vessel-dependent cancers. LncRNAs are key regulators of endothelial proliferation and play a role in angiogenesis.

**TRANSLATIONAL OUTLOOK:** Future research should examine the potential role of interventions that increase or normalize lncRNA expression and cell type–specific function to modulate angiogenesis in patients with myocardial infarction, peripheral arterial disease, or ischemic stroke.

## References

[1] Boon R.A., Iekushi K., Lechner S. (2013). MicroRNA-34a regulates cardiac ageing and function. Nature.

[2] Ucar A., Gupta S.K., Fiedler J. (2012). The miRNA-212/132 family regulates both cardiac hypertrophy and cardiomyocyte autophagy. Nat Commun.

[3] Zeller T., Keller T., Ojeda F. (2014). Assessment of microRNAs in patients with unstable angina pectoris. Eur Heart J.

[4] Janssen H.L., Reesink H.W., Lawitz E.J. (2013). Treatment of HCV infection by targeting microRNA. N Engl J Med.

[5] Rinn J.L., Chang H.Y. (2012). Genome regulation by long noncoding RNAs. Annu Rev Biochem.

[6] Thum T., Condorelli G. (2015). Long noncoding RNAs and microRNAs in cardiovascular pathophysiology. Circ Res.

[7] Volders P.J., Helsens K., Wang X. (2013). LNCipedia: A database for annotated human lncRNA transcript sequences and structures. Nucleic Acids Res.

[8] Mattick J.S., Rinn J.L. (2015). Discovery and annotation of long noncoding RNAs. Nat Struct Mol Biol.

[9] Uchida S., Dimmeler S. (2015). Long noncoding RNAs in cardiovascular diseases. Circ Res.

[10] Klattenhoff C.A., Scheuermann J.C., Surface L.E. (2013). Braveheart, a long noncoding RNA required for cardiovascular lineage commitment. Cell.

[11] Grote P., Wittler L., Hendrix D. (2013). The tissue-specific lncRNA fendrr is an essential regulator of heart and body wall development in the mouse. Dev Cell.

[12] Han P., Li W., Lin C.H. (2014). A long noncoding RNA protects the heart from pathological hypertrophy. Nature.

[13] Li K., Blum Y., Verma A. (2010). A noncoding antisense RNA in tie-1 locus regulates tie-1 function in vivo. Blood.

[14] Bell R.D., Long X., Lin M. (2014). Identification and initial functional characterization of a human vascular cell-enriched long noncoding RNA. Arterioscler Thromb Vasc Biol.

[15] Yan B., Yao J., Liu J.Y. (2015). lncRNA-MIAT regulates microvascular dysfunction by functioning as a competing endogenous RNA. Circ Res.

[16] Michalik K.M., You X., Manavski Y. (2014). Long noncoding RNA MALAT1 regulates endothelial cell function and vessel growth. Circ Res.

[17] Kumarswamy R., Bauters C., Volkmann I. (2014). Circulating long noncoding RNA, LIPCAR, predicts survival in patients with heart failure. Circ Res.

[18] Vausort M., Wagner D.R., Devaux Y. (2014). Long noncoding RNAs in patients with acute myocardial infarction. Circ Res.

[19] Carmeliet P., Dor Y., Herbert J.M. (1998). Role of HIF-1alpha in hypoxia-mediated apoptosis, cell proliferation and tumour angiogenesis. Nature.

[20] Yang F., Zhang H., Mei Y., Wu M. (2014). Reciprocal regulation of HIF-1alpha and lincRNA-p21 modulates the Warburg effect. Mol Cell.

[21] http://www.lncipedia.org/db/search?search_id=LINC00323&search_source=all&search_chromosome=-&search_start=&search_end=&search_keyword=&search_seq=&phylocsf_score_cutoff=. Available at: lncipedia.org. Accessed January 7, 2015.

[22] http://www.ensembl.org/Homo_sapiens/Gene/Summary?db=core;g=ENSG00000226496;r=21:41141493-41148133. Available at: ensembl.org. Accessed January 7, 2015.

[23] Li J.H., Liu S., Zhou H., Qu L.H., Yang J.H. (2014). starBase v2.0: decoding miRNA-ceRNA, miRNA-ncRNA and protein-RNA interaction networks from large-scale CLIP-seq data. Nucleic Acids Res.

[24] Yang J.H., Li J.H., Shao P., Zhou H., Chen Y.Q., Qu L.H. (2011). starBase: a database for exploring microRNA-mRNA interaction maps from argonaute CLIP-seq and degradome-seq data. Nucleic Acids Res.

[25] Kim J., Kang Y., Kojima Y. (2013). An endothelial apelin-FGF link mediated by miR-424 and miR-503 is disrupted in pulmonary arterial hypertension. Nat Med.

