# Supplementary material for: Development of Long Noncoding RNA-Based Strategies to Modulate Tissue Vascularization
Source: J Am Coll Cardiol. 2015 Nov 3;66(18):2005–15. doi: 10.1016/j.jacc.2015.07.081 (PMC4631810; doi:10.1016/j.jacc.2015.07.081)
Supplement: Online Data [file mmc1.pdf]

SUPPLEMENTAL MATERIAL

Cell culture (HUVEC, HCAEC, Ea.Hy926, HEK293T)
Human Umbilical Vein Endothelial Cells (HUVECs) and Human Coronary Artery Endothelial Cells (HCAECs) were purchased from Lonza and cultured in EBM-2 (Lonza) supplemented with hEGF, hydrocortisone, VEGF, hFGF-B, R^3-IGF-1, ascorbic acid, gentamicin/amphotericin-B and 10% FBS under standard cell culture conditions (37°C, 5% CO_2). The HUVEC-derived cell line Ea.Hy926 was purchased from ATTC/LGC-Standards and cultured in DMEM media (Gibco, Invitrogen) supplemented with 10% FBS and 1% Pen/Strep under standard cell culture conditions. HEK293T cells were cultured in DMEM media (Gibco, Invitrogen) supplemented with 10% FBS and 1% Pen/Strep under standard cell culture conditions. For hypoxia experiments, cells were exposed to low oxygen (0.2% O_2) under standard cell culture conditions. Cells were usually pooled from various donors.

Transfection experiments (siRNA/LNA™ GapmeR/ miRVana™)
Transient transfection of cells was performed at a confluence of 60-70% one day after seeding. Final concentration of oligonucleotides was 1 nM for siRNA and 10 nM for GapmeR. Oligonucleotides that were used for transfection experiments were as following: siRNA LINC00323-003 (GCACUGUGUGCAGUUAGCU, MWG Biotech), control siRNA (non-specific control duplex 47% GC content, MWG Biotech), LNA™ GapmeR LINC00323-003 against exon 2 (custom design, in vitro standard grade, Exiqon), control LNA™ GapmeR (negative control A, in vitro standard grade, Exiqon), control LNA™ GapmeR-FAM (negative control A, in vivo ready, 5´-FAM labelled, Exiqon), LNA™ GapmeR MIR503HG/LNC-PLAC1-1 against exon 3 (custom design, in vitro standard grade, Exiqon), miRVana™ mimic hsa-miR-503, hsa-miR-424 and control miRVana™ mimic (Life Technologies). siRNAs, GapmeRs or miRVana™ mimics and Lipofectamine 2000 (Invitrogen) were incubated separately with Opti-MEM I (Invitrogen) for 5 min, then mixed and incubated for 20 min. Serum-free conditions were applied for the transfection reaction and after 4 h medium was renewed. Cells were harvested and analysed after 48 h.

Real-time PCR analysis
Total RNA isolation was performed with TriFast reagent (Peqlab) or by the use of miRNeasy-Kit (Qiagen) according to the manufacturer's protocol. To separate cytoplasmic and nuclear RNA, cells were first lysed. Cytoplasmic and nuclear fractions were obtained by centrifugation and processed with TriFast reagent (Peqlab) for RNA isolation. To check purity of isolated nuclear RNA, lncRNA Xist (One-Step RT-PCR with QuantiFast probe assay, Qiagen) was detected. For gene expression analysis, RNA was reverse transcribed (mRNA oligo dT-primed, LINCRNA and 18S rRNA random primed) with iScript Select cDNA Synthesis Kit (Bio-Rad) and Real-time-PCR analysis was performed using iQ SYBR Green Supermix (Bio-Rad). Primer sequences were as following: LINC00323-001: forward 5´-TCCGTGAAATGAATCCATCA-3´ // reverse 5´-TCCATTCCAGTGGAGACACA-3´, LINC00323-002: forward 5´- AAGACCAGGAAGTGAGGTGAGG-3´ // reverse 5´-GCTCGGTGTTGGAAAGCAAACC-3´, LINC00323-003: forward 5´-TAGGCTTAGGGCATGAGGAG-3´ // reverse 5´-AAAAGAGAAGGGGAGGATGC-3´, MIR503HG: forward 5´- AGGTAGAAGGTGGGGTCTGC-3´ // reverse 5´-ACTGGAGGAAGCCGGATG-3´, p21: forward 5´-GCAGACCAGCATGACAGATTTC-3´ // reverse 5´-GGATTAGGGCTTCCTCTTGGA-3´, GAPDH: forward 5´-CCAGGCGCCCAATACG-3´ // reverse 5´-CCACATCGCTCAGACACCAT-3´, 18 S rRNA: forward 5´-AGTCCCTGCCCTTTGTACACA-3´ // reverse 5´-GATCCGAGGGCCTCACTAAAC-3´. Primers for human THBS1, CDK1/2 and VEGFb were purchased QuantiTect (Qiagen) primer sets. To analyze human miRNA-503, pri-miRNA-503, miRNA-424, pri-miRNA-424 expression, two step RT-PCR primer sets from Applied Biosystems were used according to the manufacturer´s protocol. RNU48 served as a housekeeping control.

Western Blotting experiments
10-30 µg of total protein were separated by SDS–PAGE, transferred to PVDF membrane and analyzed by western blotting using standard protocols. The following antibodies were used to detect antigens: SIRT1 (Cell Signaling, #8469), GATA2 (Abcam, ab22849), phospho-ERK-

1/2/ERK-1/2 (Cell Signaling, #9101/02), HMOX-1 (R&D Systems, AF3776), p27 (Abcam, ab32034) and GAPDH (Abcam, ab8245). Luminol reagent was used to detect the signals on the membrane using X-ray films (Kodak). Band intensity was calculated applying ImageJ software.

Immunoprecipitation (IP) experiments

Ea.Hy926 cells underwent IP using mouse control IgG (Santa Cruz, sc-2025) and mouse eIF4A3 IgG (Merck-Millipore, #05-1527). For IP, antibodies were coupled to Dynabeads™ (Invitrogen). 10 million cells were used for IP and separated for control and eIF4A3 pulldown group. Pulldown of proteins was performed at 4°C for 1 h. After extensive washing steps, total RNA and protein fraction was taken from magnetic beads and used for further analysis (qPCR, Western Blot).

Proliferation studies

Cells were seeded in 96-well cell culture plates and transfected liposomally with siRNA/ LNA™ GapmeR one day after seeding. To measure proliferative capacity in siRNA or LNA™ GapmeR-modulated cells, a WST-1 proliferation assay (Roche) was applied. 48 h after transfection medium was changed and replaced by medium containing WST-1 reagent according to the manufacturer´s instructions. Finally, WST-1 absorbance was measured at 450 nm. Additionally, a colorimetric BrdU ELISA kit from Roche (#11647229001) was applied. Standard procedures were performed according to manufacturers´ instructions.

Capillary tube formation assay

The ability of HUVECs to form capillaries was analyzed on growth factor reduced Matrigel™ (BD Bioscienes). Therefore, siRNA/ LNA™ GapmeR-modulated cells were seeded at a density of 15.000 cells/8well chamber slide. Capillary formation was monitored 4-6 h after seeding and quantified as cumulative capillary (tube) length with NIS-Elements BR software (Nikon).

Scratch wounding migration assay

The migration capacity of siRNA-/LNA™ GapmeR-modulated cells was analysed by the use of culture-inserts (Ibidi). Therefore, 15.000 cells (HUVECs) or 30.000 cells (Ea.Hy926) were placed to each side of culture-inserts 24 h after transfection. 24 h later, plastic patches were removed and microscopic images were captured after 0, 4, 6 and 24 hours. Wound area was calculated by the use of NIS-elements BR software (Nikon). Migration index was calculated by the formula: area (0 h) – area (6 h)/area (0 h).

Analysis of binucleic cells

siRNA LINC00323-003 modulated cells and controls were 4% PFA-fixed after 48 h of transfection. DAPI-staining was performed afterwards to count the number of binucleic and total cells via the use of NIS-elements BR software (Nikon).

Apoptosis staining

Liposomally transfected cells were subjected to Annexin V/7-AAD staining applying FlowCellect Annexin Red Kit (Merck Millipore) and analyzed on a Guava easyCyte (Merck Millipore). Apoptotic cells were considered to be Annexin V positive and 7-AAD negative. In parallel to apoptosis detection, cell size was determined after lncRNA modulation.

Generation of stable lncRNA-overexpressing Ea.Hy926 cell lines

The endothelial cell line Ea.Hy926 was used to generate stable cell lines overexpressing LINCRNAs LINC00323-003 and MIR503HG. For lentiviral cloning, gene synthesis for LINCRNAs with 5´-*AgeI* and 3´-*XbaI* restriction sites was done by GeneArt® (Life Technologies). Subcloning of genes was then performed in pLV+ plasmid containing a bidirectional promotor to express GFP, puromycin selection cassette and LINCRNA simultaneously. Lentiviral particles were produced in HEK293T cells with the help of additional plasmids pMDL-g/pIIE, pRSV-rev, pCMV-VSVG, harvested from supernatant after 48 and 72 h and concentrated applying Amicon Ultra Centrifugal Filter Units with a molecular weight cut-off 100.000 (Amicon). Concentrated lentivirus was transduced at 1:10.000 to Ea.Hy926 cells and reached a transduction efficacy of ~30%. Puromycin selection was done at a concentration of 1 µg/ml after 72 h of transduction and kept for 7d in minimum.

Rescue assays applying lentivirus
Cells were transduced with GATA2 lentivirus at day of seeding and transfected with siRNA/GapmeR against lncRNA 24 h afterwards. After 48 h, cells underwent functional phenotyping (e.g. BrdU-ELISA).
Microarray analysis
2 µg of total RNA from HUVECs cultured under normoxic or hypoxic conditions for 24 h was subjected to NCode™ Human Non-coding RNA microarray (Invitrogen) analysis. RNA of each group was pooled from triplicates to be hybridized on the microarray. Mircoarray analysis was performed using GeneSpring GX software.
RNA-Sequencing analysis
The resulting 318 million paired-end short reads (90bp + 90bp length) were evenly distributed across the six samples, with 51.4 million to 55.2 million per sample. The reads were mapped with a success rate of 97.5% on the hg19 genome via the STAR 2-pass method with default parameters (1). The information about potential splice junctions gathered by the first mapping pass from all sequenced libraries, were extracted and used to improve the second mapping pass. Exploiting the information of the mapped reads, RNA fragments were counted strand-specifically on the exon-level and subsequently summarized on the gene-level via featureCounts (2) using the protein-coding genes of the EnsEMBL GRCh37 (3) gene catalog for mRNA and the Incipedia 2.1 (4) catalog for lncRNAs. Genes were tested for differential expression using DESeq2 (5), comparing fragment counts of the three hypoxia libraries to the three normoxia libraries. Genes which resulted in Benjamini-Hochberg (6) adjusted p-values < 0.05 were considered significant.
Mapping of MicroArray and RNA-Seq data
To compare the NCode microarray to the RNA-Seq results, the microarray probes had to be mapped on the EnsEMBL and Incipedia gene catalogs used for fragment counting in RNA-Seq analysis. First, the genomic coordinates of the 39,150 probe were transferred from the human genome assembly hg18 to hg19 (7) using liftover (8) with a success rate of more than 99.9%. Strand-specific overlaps between transferred probes and exons of protein-coding genes of the EnsEMBL gene catalog or the Incipedia 2.1 lncRNA catalog were identified via bedtools (9). This resulted in 17,444 probes mapping on 10,582 protein-coding EnsEMBL genes and 4,425 probes mapping on 3,594 Incipedia 2.1 lncRNAs.Since more than 4,500 protein-coding genes and more than 500 lncRNAs were covered by more than one probe, the median of log2 fold changes was calculated to get one value of the microarray for each gene.
GEO (Gene expression omnibus) accession numbers
Microarray and RNA-Seq datasets were uploaded to GEO at NCBI and grouped under a SuperSeries GSE70335.
lncRNA homology search
ECR browser was used to check for homology of identified lncRNAs. For this we used the default settings given (e.g. evolutionary conserved regions ECRs, minimum length 100 bp, minimum identity 70%) (see http://ecrbrowser.dcode.org/).
Cell culture of human induced pluripotent stem cells (hiPSCs)
HiPS cell line was derived by reprogramming dermal fibroblasts with the 4 factors OCT4, SOX2, KLF4 and c-MYC (Dr. Moretti, Munich) as previously described (Park et al., Moretti et al. 2010). Pluripotency was confirmed by in vivo teratoma formation. HiPSCs were cultured under feeder-free conditions on Geltrex® in CF1-MEF conditioned medium. Confluent cultures were passaged with 0.5 mM EDTA (5 min, 37 °C).
hiPS-cardiomyocyte differentiation
Confluent layers of hiPS cell colonies were digested with EDTA (0.5 mM; Roth 8043.2; 1 ml/10 cm^2) for 10 minutes. EDTA was removed and PBS was added (1 ml/10 cm^2). After washing, the hiPS cells were re-suspended in EB formation medium (CF1-MEF conditioned medium; 4 mg/ml polyvinyl alcohol, Sigma-Aldrich P8136; 10 µM Y-27632, biorbyt orb60104; 30 ng/ml basic FGF, R&D systems 233-FB) to a density of $30x10^6$cells/100 ml. Embryoid bodies (EBs) were generated by seeding the single cell suspension into spinner flasks ($30x10^6$ /100 ml; Integra 182 101) and incubating overnight with 40 rounds per minute rotation under hypoxic conditions (37 °C, 5% CO_2, 5% O_2, 90% humidity). After 24 hours EBs

were collected and EB volume was estimated. Pelleted EBs were re-suspendend in Mesoderm induction medium (RPMI 1640, Gibco 21875; 4 mg/ml polyvinylalcohol, Sigma-Aldrich P8136; 0.1% lipid mix, Sigma-Aldrich L5146; 250 µM phosphoascorbat, Sigma-Aldrich 49752; 1% Transferrin-Selenium; 1% Penicillin/Streptomycin, Gibco 15140; 10 µM Y-27632, biorbyt orb60104; 10 ng/ml BMP-4, R&D systems 314-BP; 3 ng/ml Activin-A, R&D systems 338-AC; , 5 ng/ml basic FGF, R&D systems 233-FB). Pluronic-coated ULA-cell culture flasks (ULA-CCF) were washed twice with PBS. EBs were transferred to the ULA-CCF with a density of 100 µl EB volume/35 ml of Mesoderm induction medium per T175 cell culture flask. EBs were incubated for 3 days with daily media change under hypoxic conditions (37 °C, 5% CO_2, 5% O_2, 90% humidity). For cardiomyocyte differentiation, EBs were transferred to Cardiac differentiation medium I (RPMI 1640, Gibco 21875; 0,1% lipid mix, Sigma-Aldrich L5146; 250 µM phosphoascorbat, Sigma-Aldrich 49752; 1% Transferrin-Selenium; 1% Penicillin/Streptomycin, Gibco 15140; 1 µM Y-27632, biorbyt orb60104; 100 nM 4-(cis-endo-1,3-dioxooctahydro-2H-4,7-methanoisoindol-2-yl)-N-(quinolin-8-yl)-transcyclohexylcarbox-amide(DS-I-7)) and seeded into Geltrex®-coated cell culture flasks at a density of 100 µl EB volume/ 25 ml Cardiac differentiation medium I. EBs were incubated for 3 days with daily medium change under standard culture conditions (37 °C, 5% CO_2, 20% O_2, 90% humidity). On day 7 medium was changed to cardiac differentiation medium II (RPMI 1640, Gibco 21875; 500 µM 1-Thioglycerol, Sigma-Aldrich M6145; 10 mM HEPES, Roth, 9105.4; 0.5% Penicillin/Streptomycin, Gibco, 15140; 1 µM Y-27632, biorbyt orb60104; 2% B27 plus insulin, Gibco, 17504-044; 100 nM DS-I-7). EBs were incubated for additional 4 days with daily medium change under standard culture conditions. For cardiac maturation, DS-I-7 was omitted and EBs were cultivated for another 3 days under standard culture conditions. On day 14 of cardiac differentiation the EBs were dissociated with collagenase II (200 U/ml, Worthington, LS004176 in HBSS minus Ca^{2+}/Mg^{2+}, Gibco, 14175-053) for 3.5 hours at 37 °C. Single cell suspensions of hiPS-cardiomyocytes were directly subjected to EHT generation.

Generation and culture of human Engineered Heart Tissue (EHT)

Teflon spacers and silicone racks with silicone posts were manufactured as previously described (Hansen et al., 2010). Silicone racks were produced by Siltec GmbH & Co KG. For EHT generation, the following reconstitution mix was prepared on ice: 5 x 10^5 hiPS-cardiomyocytes/ EHT (1.39 x 10^7 cells/ml), 2 x 10^5 GFP-expressing HUVECs/EHT (1 x 10^7 cells/ml), 5 mg/ml bovine fibrinogen (stock solution: 200 mg/ml fibrinogen plus aprotinin 100 µg/ml in NaCl 0.9%; Sigma F4753), 10% Matrigel (BD Bioscience 356235). To ensure isotonic conditions, 2x Dulbecco´s modified Eagle medium (DMEM) was added to match the volumes of fibrinogen and thrombin stock (100 U/ml; Sigma T7513). Casting molds were prepared as previously described (10). For each EHT 97 µl reconstitution mix was briefly mixed with 3 µl thrombin and pipetted into an agarose slot. After fibrinogen polymerization in a humidified cell culture incubator (1.5 h), the racks were transferred to 24-well cell culture plates containing EHT-culture medium. EHTs were cultured in a 37°C, 7% CO_2, 40% O_2 humidified cell culture incubator with a medium consisting of DMEM (Biochrom F0415), 10% heat-inactivated horse serum (Gibco 26050), 1% penicillin/streptomycin (Gibco 15140), insulin (10 µg/ml; Sigma I9278) and aprotinin (33 µg/ml; Sigma A1153).

Transfection of human EHTs

Transfection of siRNA LINC00323-003 and LNA™ GapmeR MIR503HG was performed with Turbofect (Thermo Scientific, R0531) according to the manufacturer´s instructions. EHTs were transfected on day 5, 10 and 15 of EHT culture with 1 nM siRNA LINC00323-003 or 10 nM of GapmeR LNC-PLAC1-1. As controls, control-siRNA/GapmeR were transfected. EHTs were prepared for immunohistochemistry on day 20.

Immunohistochemistry and image analysis

EHTs were fixed in 4% formaldehyde overnight, washed in TBS, paraffin embedded and sectioned transversely in 4 µm slices. Conditions for immunohistochemistry were: mouse anti-dystrophin monoclonal antibody (Millipore, MAB1645), antigen retrieval: 60 min in EDTA-buffer, pH 8.0; rabbit anti-GFP polyclonal antibody (Abcam, ab290), antigen retrieval: 60 min in citrate-buffer, pH 6.0. All antibodies were visualized with the multimer-technology based UltraView Universal DAB Detection Kit (Roche). GFP-expressing cells were quantified in 3

EHTs per group and 3 levels per EHTs. Microscopic images were taken on an Axioskop 2 microscope (Zeiss).

Statistical Analysis

For statistical analysis, GraphPad Prism (Version 4) was applied. In case of two groups, unpaired t-test was performed. Error bars in graphs indicate standard error of the mean (SEM). One experiment contained 3 replicates per group. Pearson correlation and Fisher exact test were performed in R version 3.1.2 (11). Asterisk mean: * = $p < 0.05$; ** = $p < 0.01$; *** = $p < 0.001$.

References

1. Dobin A, Davis CA, Schlesinger F*, et al*. STAR: Ultrafast universal RNA-seq aligner. Bioinformatics. 2013;29:15-21.
2. Liao Y, Smyth GK, Shi W. featureCounts: An efficient general purpose program for assigning sequence reads to genomic features. Bioinformatics. 2014;30:923-30.
3. Flicek P, Amode MR, Barrell D*, et al*. Ensembl 2014. Nucleic acids research. 2014;42:D749-55.
4. Volders PJ, Helsens K, Wang X*, et al*. LNCipedia: A database for annotated human lncRNA transcript sequences and structures. Nucleic Acids Res. 2013;41:D246-51.
5. Love MI, Huber W, Anders S. Moderated estimation of fold change and dispersion for RNA-seq data with DESeq2. Genome biology. 2014;15:550.
6. Hochberg Y, Benjamini Y. More powerful procedures for multiple significance testing. Statistics in medicine. 1990;9:811-8.
7. Rhead B, Karolchik D, Kuhn RM*, et al*. The UCSC genome browser database: Update 2010. Nucleic acids research. 2010;38:D613-9.
8. Hinrichs AS, Karolchik D, Baertsch R*, et al*. The UCSC genome browser database: Update 2006. Nucleic acids research. 2006;34:D590-8.
9. Quinlan AR, Hall IM. BEDTools: A flexible suite of utilities for comparing genomic features. Bioinformatics. 2010;26:841-2.
10. Hansen A, Eder A, Bonstrup M*, et al*. Development of a drug screening platform based on engineered heart tissue. Circ Res. 2010;107:35-44.
11. Team RC. R: A language and environment for statistical computing. . 2014.

Supplemental Figure Legends

Supplemental Figure I: (A, B) Comparison of microarray-derived and RNA-sequencing results. RNA-Seq analysis for long non-coding RNA of HUVECs cultured in normoxic or hypoxic conditions (*n* = 3 experiments). For microarray analysis, total RNA was pooled from *n* = 3 experiments. (A) Log2 fold changes of lncRNAs between hypoxic and normoxic conditions were significantly correlated in the microarray and RNA-Seq data. (B) The log2 fold changes of protein-coding genes were even stronger correlated. (C) LINC00323 transcripts #1 and #2 are upregulated after hypoxia. Validation experiments were performed in fractionated RNA (total, nuclear and cytoplasmic) from HUVEC in normoxic and hypoxic culture conditions by qRT-PCR. LINC00323-1/2 expression levels are increased after hypoxia (*n* = 2-6 experiments). (D) Quality check of RNA fractionation. Total RNA was fractionated to nuclear and cytoplasmic samples and underwent detection of nuclear-specific lncRNA XIST (*n* = 4 experiments). (E) LncRNA expression in human cardiac fibroblasts (HCFs) and human aortic smooth muscle cells (HASMCs). HCFs or HASMCs were subjected to 24 h of hypoxia and lncRNA expression was determined afterwards via qRT-PCR (*n* = 3 experiments). All experiments were conducted with three technical replicates.* = $p < 0.05$, ** = $p < 0.01$

Supplemental Figure II: (A) Efficacy of LINC00323-003 knockdown. Transgenic Ea.Hy926 cells overexpressing LINC00323-003 were transfected applying 100 nM siRNA or 10 nM of GapmeR for 24 h before LINC00323-003 expression was determined via qRT-PCR (*n* = 3 experiments (siRNA) or *n* = 3 replicates with GapmeR). (B) Subcellular localization of GapmeR. HUVECs were liposomally transfected applying 50 nM of GapmeR-FITC highlighting nuclear localization of GapmeR. BF = brightfield; scale bar = 100 µm (C) siRNA LINC00323-003 titration experiments. Transient knockdown of LINC00323-003 triggering viability defects in different siRNA dosing measured by WST1 viability assay in HUVECs (*n* = 3 experiments). (D) GapmeR transfection towards LINC00323-003 triggers proliferative defects in HUVECs determined by BrdU-ELISA (*n* = 3 experiments). (E) The acquisition of binucleic vs. total cells reveals more binuclei number in LINC00323-003-deficient HUVECs (*n* = 3 experiments). All experiments were conducted with three technical replicates. * = $p < 0.05$, ** = $p < 0.01$, *** = $p < 0.001$

Supplemental Figure III: (A) Loss of LINC00323-003 increases cell size. Liposomal transfection of 1 nM siRNA LINC00323-003 in HUVECs causes increase in cell size measured by FACS (*n* = 5 experiments). (B) LINC00323-003 knockdown decreases CDK1/2 expression. HUVECs were transfected with 1 nM siRNA LINC00323-003 for 48 h and CDK1/2 expression was determined afterwards by qRT-PCR (*n* = 3 experiments). (C) Endothelial wound healing is impaired by the loss of LINC00323-003. Liposomal transfection of 1 nM siRNA LINC00323-003 impairs endothelial wound healing after initial scratch wound (*n* = 3 experiments). (D) LINC00323-003 knockdown decreases VEGFb and increases THBS1 expression. HUVECs were transfected with 1 nM siRNA LINC00323-003 for 48 h and pro-/anti-angiogenic VEGFb, THBS1 expression was determined afterwards by qRT-PCR (*n* = 3 experiments). (E) Cell cycle inhibitor p21 (mRNA level) is increased in LINC00323-003 knockdown HUVECs (*n* = 3 experiments). (F) ERK growth factor signalling is impaired in siRNA LINC00323-003 transfected HUVECs (*n* = 4 experiments). (G) GATA2 supplementation via lentivirus can revert the phenotype of LINC00323-003 knockdown. BrdU-ELISA was performed to monitor proliferative capacity (*n* = 4 experiments). (H) LINC00323-003 knockdown decreases HCAEC proliferation. Liposomal-based siRNA transfection towards LINC00323-003 triggers proliferative defects in HCAECs determined by BrdU-ELISA (*n* = 3 experiments). All experiments were conducted with three technical replicates.* = $p < 0.05$, ** = $p < 0.01$, *** = $p < 0.001$

Supplemental Figure IV: (A) Enhanced miR-503 expression decreases WST1 viability. HUVECs were liposomally transfected with 100 nM of miR-503 for 48 h and WST1 assay was performed afterwards (*n* = 3 experiments). (B) Increase in cell cycle inhibitor p21 expression detected by qPCR in MIR503HG deficient HUVECs (*n* = 3 experiments). (C) MIR503HG knockdown is not inducing apoptosis. HUVECs were liposomally transfected with 10 nM of GapmeR against MIR503HG for 48 h, underwent Annexin-V/PI staining and FACS analysis afterwards (*n* = 3 experiments). (D) MIR503HG repression reduces miR-503 expression. HUVECs were liposomally transfected with 10 nM of GapmeR against MIR503HG for 48 h and underwent qRT-PCR analysis for miR-503 and RNU48 afterwards (*n* = 3 experiments). (E) Enhanced miR-503 expression has no effect on MIR503HG expression. HUVECs were liposomally transfected with 30 nM of miR-503 for 72 h and WST1 assay was performed afterwards (*n* = 3 experiments). (F) GapmeR against LINCRNA MIR503HG reduces capillary tube formation. HUVECs were liposomally transfected with 10 nM of GapmeR against MIR503HG for 48 h and capillary tube formation was monitored on matrigel. Scale bar = 500 µm (*n* = 5 experiments). (G) MIR503HG knockdown has minor effects in HCAECs. HCAECs were liposomally transfected with 10 nM of GapmeR against MIR503HG for 48 h and BrdU-incorporation rate was determined by ELISA (*n* = 3 experiments). All experiments were conducted with three technical replicates.* = $p < 0.05$, ** = $p < 0.01$, *** = $p < 0.001$

Supplemental Figure V: (A) LINCRNA overexpression in Ea.Hy926 cell line. Ea.Hy926 cells were transduced with control or LINCRNA (LINC00323-003 or MIR503HG) lentivirus to

generate a stable cell line. LINCRNA overexpression rate for LINC00323-003 and I MIR503HG was monitored afterwards via qRT-PCR. Brightfield (BF) picture and GFP expression of control transgenic Ea.hy926 is shown exemplary. Scale bar = 100 µm (*n* = 3 experiments). (B) LINCRNA LINC00323-003 overexpression in Ea.Hy926 cell line increases HMOX-1. Transgenic Ea.Hy926 with stable overexpression of LINC00323-003 have increased expression of cytoprotective HMOX-1 (*n* = 3 experiments). (C) Migration index and scratch wound closure is improved in transgenic Ea.Hy926 cells overexpressing LINC00323-003 and MIR503HG (*n* = 5 experiments). All experiments were conducted with three technical replicates. * = $p < 0.05$, ** = $p < 0.01$, *** = $p < 0.001$

Supplemental Figure I

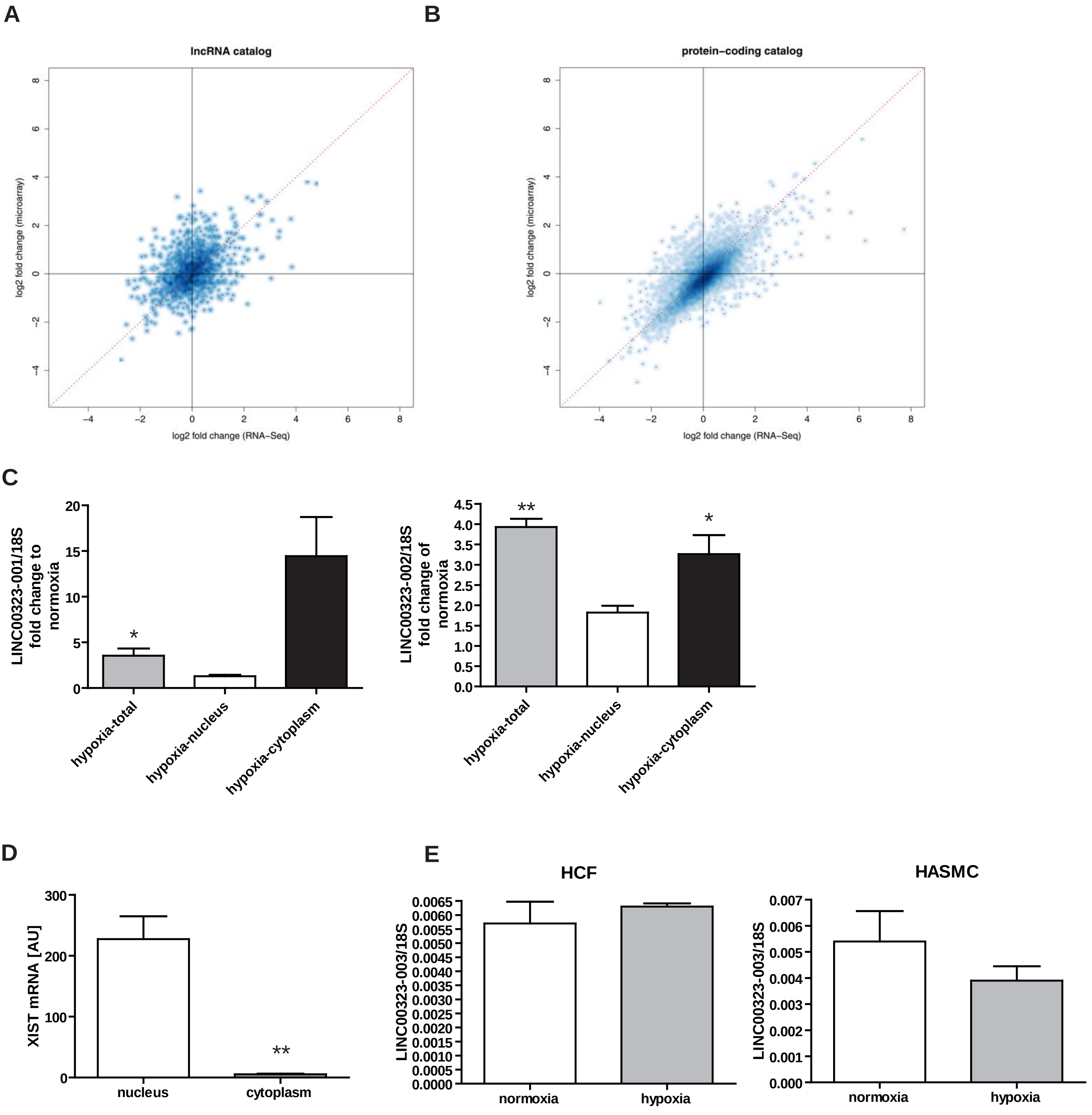

Supplemental Figure I: (A, B) Comparison of microarray-derived and RNA-sequencing results. RNA-Seq analysis for long non-coding RNA of HUVECs cultured in normoxic or hypoxic conditions (*n* = 3 experiments). For microarray analysis, total RNA was pooled from *n* = 3 experiments. (A) Log2 fold changes of lncRNAs between hypoxic and normoxic conditions were significantly correlated in the microarray and RNA-Seq data. (B) The log2 fold changes of protein-coding genes were even stronger correlated. (C) LINC00323 transcripts #1 and #2 are upregulated after hypoxia. Validation experiments were performed in fractionated RNA (total, nuclear and cytoplasmic) from HUVEC in normoxic and hypoxic culture conditions by qPCR. LINC00323-1/2 expression levels are increased after hypoxia (*n* = 2-6 experiments). (D) Quality check of RNA fractionation. Total RNA was fractionated to nuclear and cytoplasmic samples and underwent detection of nuclear-specific lncRNA XIST (*n* = 4 experiments). (E) LncRNA expression in human cardiac fibroblasts (HCFs) and human aortic smooth muscle cells (HASMCs). HCFs or HASMCs were subjected to 24 h of hypoxia and lncRNA expression was determined afterwards via qRT-PCR (*n* = 3 experiments). All experiments were conducted with three technical replicates. * = $p < 0.05$, ** = $p < 0.01$

Supplemental Figure II

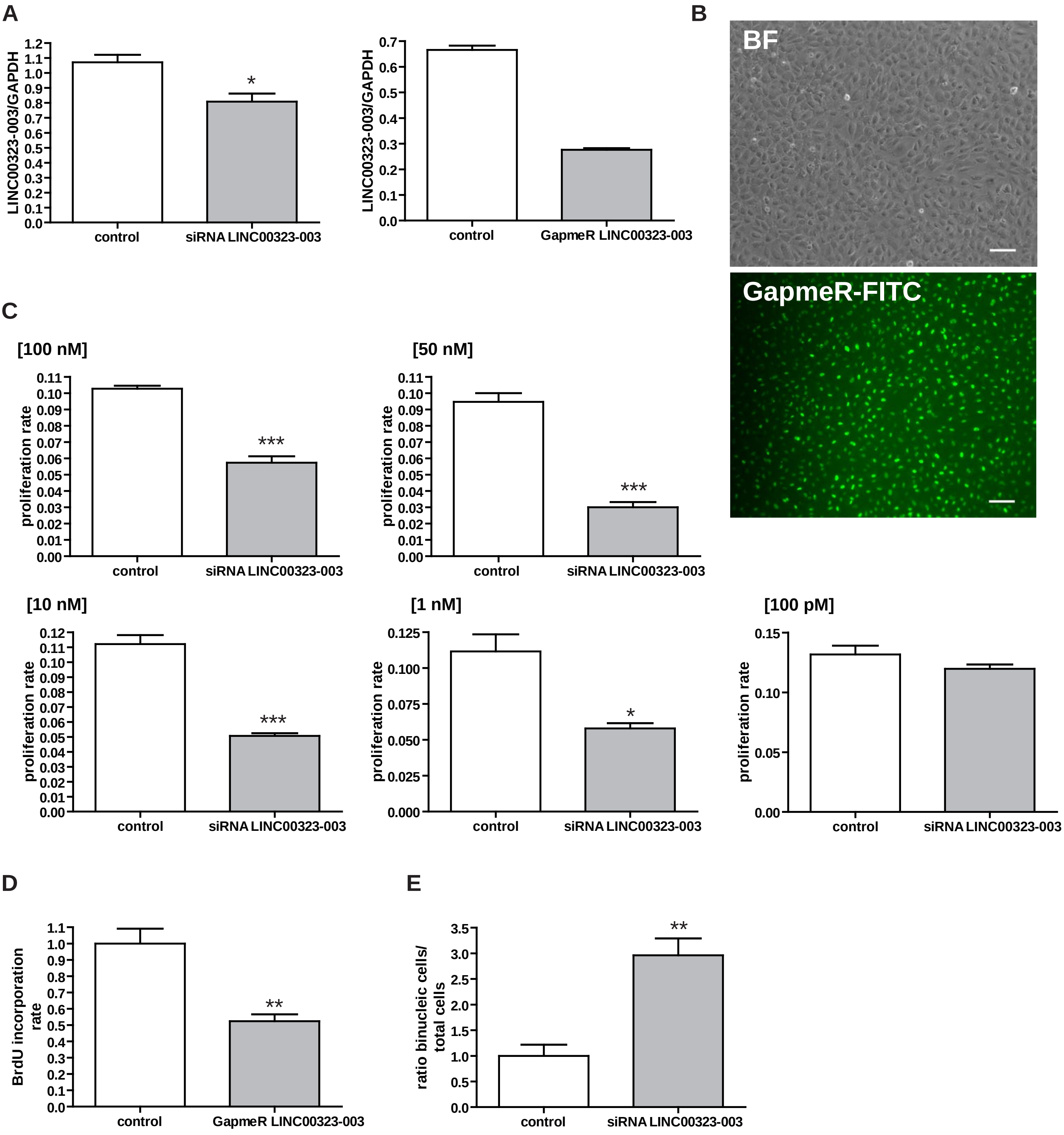

Supplemental Figure II: (A) Efficacy of LINC00323-003 knockdown. Transgenic Ea.Hy926 cells overexpressing LINC00323-003 were transfected applying 100 nM siRNA or 10 nM of GapmeR for 24 h before LINC00323-003 expression was determined via qRT-PCR. (*n* = 3 experiments (siRNA) or *n* = 3 replicates with GapmeR). (B) Subcellular localization of GapmeR. HUVECs were liposomally transfected applying 50 nM of GapmeR-FITC highlighting nuclear localization of GapmeR. BF = brightfield; scale bar = 100 µm (C) siRNA LINC00323-003 titration experiments. Transient knockdown of LINC00323-003 triggering viability defects in different siRNA dosing measured by WST1 viability assay in HUVECs (*n* = 3 experiments). (D) GapmeR transfection towards LINC00323-003 triggers proliferative defects in HUVECs determined by BrdU-ELISA (*n* = 3 experiments). (E) The acquisition of binucleic vs. total cells reveals more binuclei number in LINC00323-003-deficient HUVECs (*n* = 3 experiments).
All experiments were conducted with three technical replicates. * = p < 0.05, ** = p < 0.01, *** = p < 0.001

Supplemental Figure III

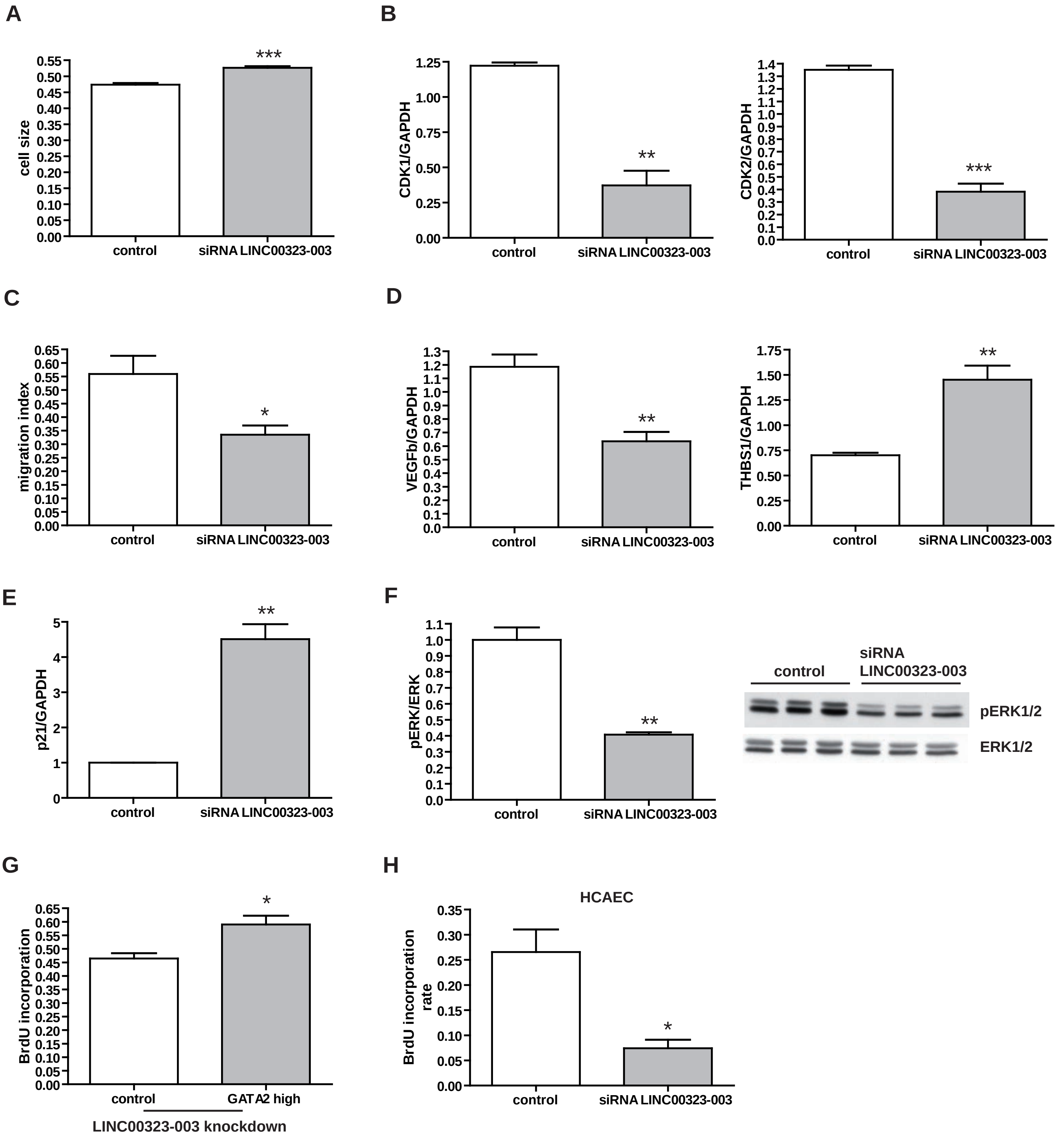

Supplemental Figure III: (A) Loss of LINC00323-003 increases cell size. Liposomal transfection of 1 nM siRNA LINC00323-003 in HUVECs causes increase in cell size measured by FACS (*n* = 5 experiments). (B) LINC00323-003 knockdown decreases CDK1/2 expression. HUVECs were transfected with 1 nM siRNA LINC00323-003 for 48 h and CDK1/2 expression was determined afterwards by qRT-PCR (*n* = 3 experiments). (C) Endothelial wound healing is impaired by the loss of LINC00323-003. Liposomal transfection of 1 nM siRNA LINC00323-003 impairs endothelial wound healing after initial scratch wound (*n* = 3 experiments). (D) LINC00323-003 knockdown decreases VEGFb and increases THBS1 expression. HUVECs were transfected with 1 nM siRNA LINC00323-003 for 48 h and pro-/anti-angiogenic VEGFb, THBS1 expression was determined afterwards by qRT-PCR (*n* = 3 experiments). (E) Cell cycle inhibitor p21 (mRNA level) is increased in LINC00323-003 knockdown HUVECs (*n* = 3 experiments). (F) ERK growth factor signalling is impaired in siRNA LINC00323-003 transfected HUVECs (*n* = 4 experiments). (G) GATA2 supplementation via lentivirus can revert the phenotype of LINC00323-003 knockdown. BrdU-ELISA was performed to monitor proliferative capacity (*n* = 4 experiments). (H) LINC00323-003 knockdown decreases HCAEC proliferation. Liposomal-based siRNA transfection towards LINC00323-003 triggers proliferative defects in HCAECs determined by BrdU-ELISA (*n* = 3 experiments). All experiments were conducted with three technical replicates. * = p < 0.05, ** = p < 0.01, *** = p < 0.001

Supplemental Figure IV

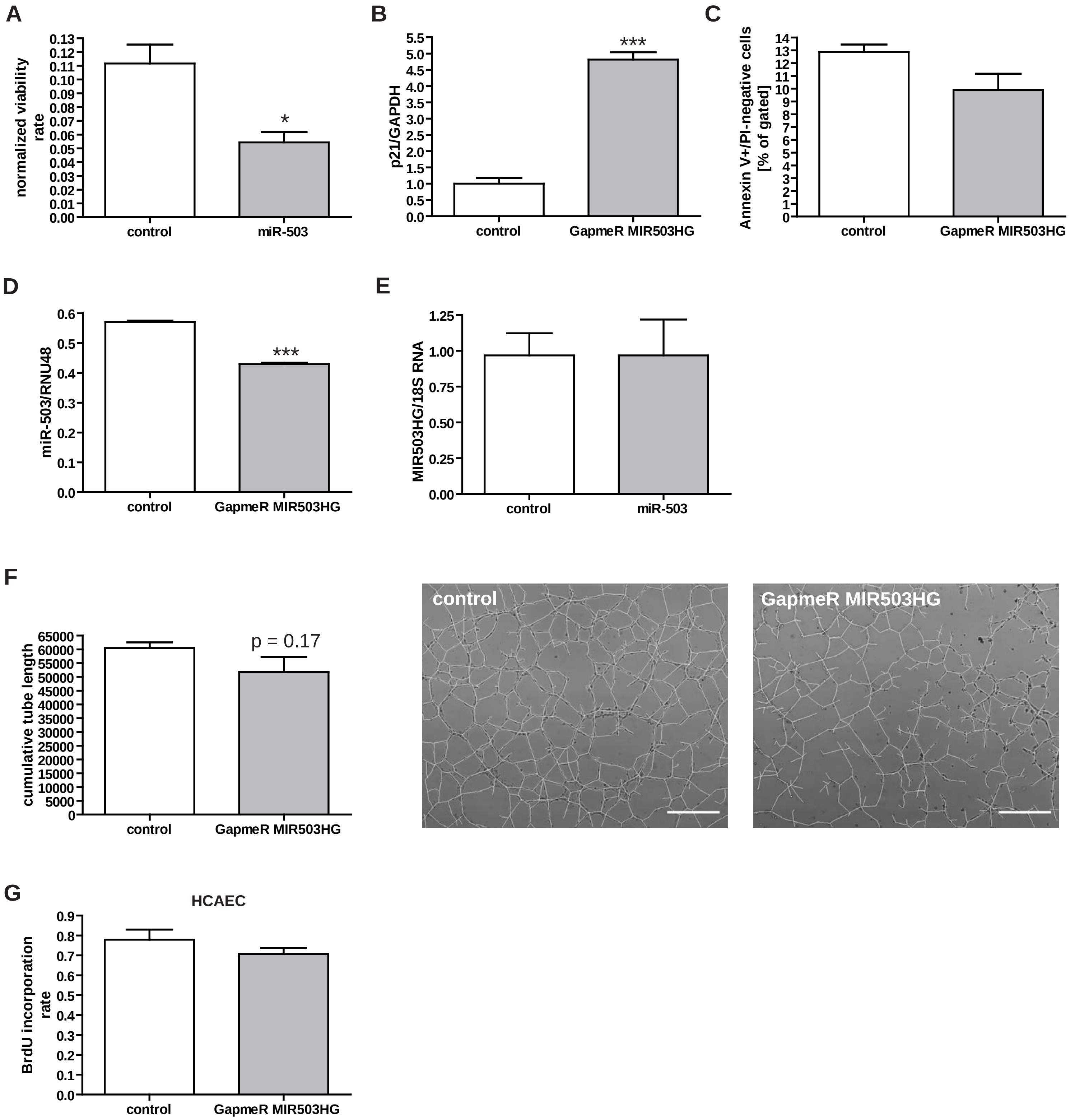

Supplemental Figure IV: (A) Enhanced miR-503 expression decreases WST1 viability. HUVECs were liposomally transfected with 100 nM of miR-503 for 48 h and WST1 assay was performed afterwards (*n* = 3 experiments). (B) Increase in cell cycle inhibitor p21 expression detected by qPCR in MIR503HG deficient HUVECs (*n* = 3 experiments). (C) MIR503HG knockdown is not inducing apoptosis. HUVECs were liposomally transfected with 10 nM of GapmeR against MIR503HG for 48 h, underwent Annexin-V/PI staining and FACS analysis afterwards (*n* = 3 experiments). (D) MIR503HG repression reduces miR-503 expression. HUVECs were liposomally transfected with 10 nM of GapmeR against MIR503HG for 48 h and underwent qRT-PCR analysis for miR-503 and RNU48 afterwards (*n* = 3 experiments). (E) Enhanced miR-503 expression has no effect on MIR503HG expression. HUVECs were liposomally transfected with 30 nM of miR-503 for 72 h and WST1 assay was performed afterwards (*n* = 3 experiments). (F) GapmeR against LINCRNA MIR503HG reduces capillary tube formation. HUVECs were liposomally transfected with 10 nM of GapmeR against MIR503HG for 48 h and capillary tube formation was monitored on matrigel. Scale bar = 500 µm (*n* = 5 experiments). (G) MIR503HG knockdown has minor effects in HCAECs. HCAECs were liposomally transfected with 10 nM of GapmeR against MIR503HG for 48 h and BrdU-incorporation rate was determined by ELISA (*n* = 3 experiments).
All experiments were conducted with three technical replicates. * = $p < 0.05$, ** = $p < 0.01$, *** = $p < 0.001$

Supplemental Figure V

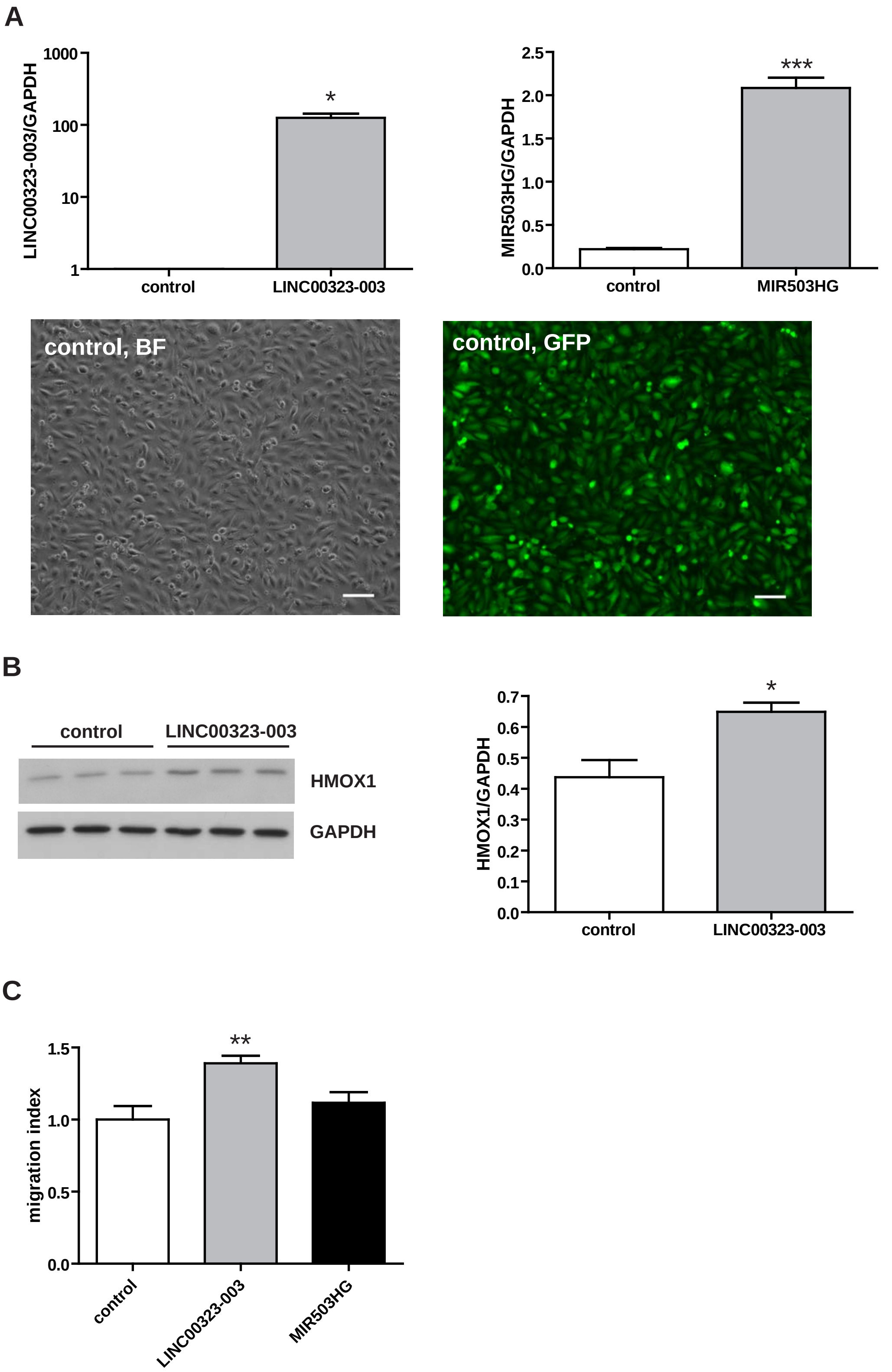

Supplemental Figure V: (A) LINCRNA overexpression in Ea.Hy926 cell line. Ea.Hy926 cells were transduced with control or LINCRNA (LINC00323-003 or MIR503HG) lentivirus to generate a stable cell line. LINCRNA overexpression rate for LINC00323-003 and MIR503HG was monitored afterwards via qPCR. Brightfield (BF) picture and GFP expression of control transgenic Ea.hy926 is shown exemplary. Scale bar = 100 µm (*n* = 3 experiments).
(B) LINCRNA LINC00323-003 overexpression in Ea.Hy926 cell line increases HMOX-1. Transgenic Ea.Hy926 with stable overexpression of LINC00323-003 have increased expression of cytoprotective HMOX-1 (*n* = 3 experiments). (C) Migration index and scratch wound closure is improved in transgenic Ea.Hy926 cells overexpressing LINC00323-003 and MIR503HG (*n* = 5 experiments). All experiments were conducted with three technical replicates.
* = $p < 0.05$, ** = $p < 0.01$, *** = $p < 0.001$
