# Supplementary material for: Development of Long Noncoding RNA-Based Strategies to Modulate Tissue Vascularization
Source: J Am Coll Cardiol. 2015 Nov 3;66(18):2005–15. doi: 10.1016/j.jacc.2015.07.081 (PMC4631810; doi:10.1016/j.jacc.2015.07.081)
Supplement: Legends for Online Tables 1 and 2 [file mmc2.docx]

**Online Table 1 (Excel file provided):** Microarray results from NCode™ Array.

**Online Supplemental Table 2 (Excel file provided): (A) Overview of protein-coding EnsEMBL genes**. For each significantly deregulated (adjusted p-value < 0.05) gene, information about the microarray experiment results (log2 fold change) and the results of the RNA-Seq experiment (mean of the normalized read counts of all 6 sequenced libraries; log2 fold change; p-value; Benjaimini-Hochberg-adjusted p-value; the normalized and raw counts for each library) is provided. The EnsEMBL gene description, the associated gene name and information about the gene panel are available. HUVEC1-3: normoxia, HUVEC4-6: hypoxia. **(B) Overview of lncipedia 2.1 lncRNAs**. For each significantly deregulated (adjusted p-value < 0.05) lncRNA, information about the microarray experiment results (log2 fold change) and the results of the RNA-Seq experiment (mean of the normalized read counts of all 6 sequenced libraries; log2 fold change; p-value; Benjaimini-Hochberg-adjusted p-value; the normalized and raw counts for each library) is provided. HUVEC1-3: normoxia, HUVEC4-6: hypoxia.
